# Acridinium 2-hy­droxy­benzoate

**DOI:** 10.1107/S160053681004345X

**Published:** 2010-10-31

**Authors:** Hossein Eshtiagh-Hosseini, Azam Hassanpoor, Masoud Mirzaei, Ali R. Salimi

**Affiliations:** a Department of Chemistry, School of Sciences, Ferdowsi University of Mashhad, Mashhad, Iran

## Abstract

In the title compound, C_13_H_10_N^+^·C_7_H_5_O_3_^−^ or (acrH)^+^(Hsal)^−^, the asymmetric unit contains one acridinium cation and one salicylate anion. The acridinium N atom is protonated and the carb­oxy­lic acid group of salicylic acid is deprotonated. Both moieties are planar, with an r.m.s. deviation of 0.0127 Å for the acr cation and 0.0235 ° for the sal anion. They are aligned with a dihedral angle of 71.68 (3)° between them. The crystal structure is stabilized by a network of inter­molecular N—H⋯O, O—H⋯O and C—H⋯O hydrogen bonds. C—H⋯π inter­actions are also present.

## Related literature

For work on mol­ecular self-association, see: Moghimi *et al.* (2005 ▶); Eshtiagh-Hosseini, Hassanpoor, Canadillas-Delgado & Mirzaei (2010 ▶); Eshtiagh-Hosseini, Mahjoobizadeh & Mirzaei (2010 ▶). For related structures, see: Gellert & Hsu (1988 ▶); Hemamalini & Fun (2010 ▶); Muthiah *et al.* (2006 ▶).
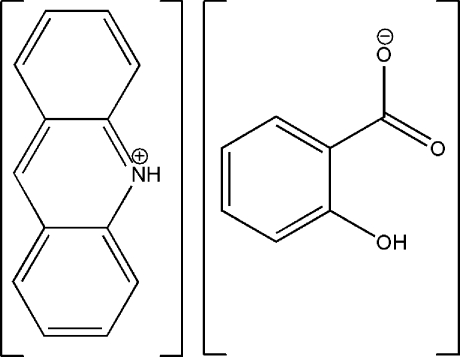


## Experimental

### 

#### Crystal data

C_13_H_10_N^+^·C_7_H_5_O_3_^−^
*M* *_r_* = 317.33
Monoclinic, 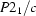

*a* = 7.128 (3) Å
*b* = 9.472 (3) Å
*c* = 22.637 (9) Å
β = 91.449 (10)°
*V* = 1527.9 (10) Å^3^
*Z* = 4
Mo *K*α radiation
μ = 0.09 mm^−1^
*T* = 100 K
0.30 × 0.25 × 0.10 mm

#### Data collection

Bruker SMART APEXII CCD area-detector diffractometer
Absorption correction: multi-scan (*SADABS*; Bruker, 2001 ▶) *T*_min_ = 0.973, *T*_max_ = 0.991
10437 measured reflections
4488 independent reflections
3161 reflections with *I* > 2σ(*I*)
*R*_int_ = 0.035

#### Refinement

*R*[*F* ^2^ > 2σ(*F* ^2^)] = 0.048
*wR*(*F* ^2^) = 0.128
*S* = 1.04
4488 reflections
241 parameters
H atoms treated by a mixture of independent and constrained refinement
Δρ_max_ = 0.37 e Å^−3^
Δρ_min_ = −0.24 e Å^−3^

### 

Data collection: *APEX2* (Bruker, 2005 ▶); cell refinement: *SAINT* (Bruker, 2005 ▶); data reduction: *SAINT*; program(s) used to solve structure: *SHELXS97* (Sheldrick, 2008 ▶); program(s) used to refine structure: *SHELXL97* (Sheldrick, 2008 ▶); molecular graphics: *ORTEP-3 for Windows* (Farrugia, 1997 ▶); software used to prepare material for publication: *WinGX* (Farrugia, 1999 ▶).

## Supplementary Material

Crystal structure: contains datablocks I, global. DOI: 10.1107/S160053681004345X/bq2237sup1.cif

Structure factors: contains datablocks I. DOI: 10.1107/S160053681004345X/bq2237Isup2.hkl

Additional supplementary materials:  crystallographic information; 3D view; checkCIF report

## Figures and Tables

**Table 1:** Hydrogen-bond geometry (Å, °) *Cg*1 is the centroid of the C2–C7 benzene ring of Hsal^−^.

*D*—H⋯*A*	*D*—H	H⋯*A*	*D*⋯*A*	*D*—H⋯*A*
N1—H1⋯O1^i^	1.05 (2)	2.49 (2)	3.100 (2)	116.4 (15)
N1—H1⋯O2^i^	1.05 (2)	1.55 (2)	2.5887 (19)	174.8 (18)
O3—H3⋯O1	1.00 (3)	1.58 (2)	2.5141 (19)	153 (2)
C10—H10⋯O1^ii^	0.93	2.49	3.294 (2)	145
C18—H18⋯O3^iii^	0.93	2.46	3.135 (2)	129
C14—H14⋯*Cg*1^iv^	0.93	2.76	3.644 (2)	159
C17—H17⋯*Cg*1	0.93	2.91	3.716 (2)	146

Symmetry codes: (i) 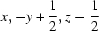; (ii) 
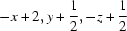
; (iii) 
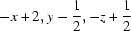
; (iv) 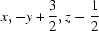.

**Fig. 1.**
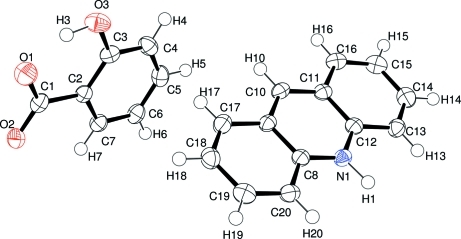
Schematic representation of asymmetric units of the title compound.

**Fig. 2.**
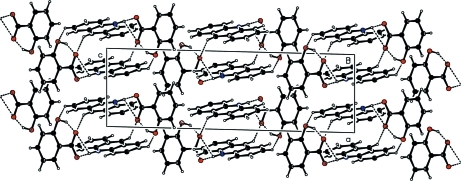
Molecular packing of the title compound with hydrogen bonding shown as dashed lines.

## supplementary crystallographic information

### Comment

Molecular self-association involves the spontaneous association of molecules into stable aggregates, joined by ion-pairing, hydrogen bonding, π–π stacking and donor–acceptor intractions (Moghimi *et al.*, 2005). Our research group recently focused on the syntheses as suitable ligands in the synthesis of metal–organic framework. For example, ion pairs have been reported between pyrazine-2,3-dicarboxylic acid with 2,4,6-triamino-1,3,5-triazin (Eshtiagh-Hosseini, Hassanpoor *et al.*, 2010) and 4-hydroxy pyridine-2,6-dicarboxylic acid bearing 2-amino pyrimidine (Eshtiagh-Hosseini, Mahjoobizadeh *et al.*, 2010). Salicylic acid is important in biological systems thus there have been several attempts to prepare proton-transfer compounds involving H_2_sal with various organic bases such as 2-amino pyridine (Gellert & Hsu, 1988), 2-amino-4,6-dimethyl primidine (Muthiah *et al.*, 2006) and 2-amino-5-chloroprimidine (Hemamalini & Fun, 2010). In this work, we reported a new proton-transfer compound obtained from salicylic acid (H_2_sal) as a proton donor and acridine (acr) as an acceptor in which acridinium N atom is protonated and carboxylic group of salicilic acid is deprotonated. The molecular structure of I, is shown in Fig. 1. The crystal structure is stabilized by a network of intermolecular N—H···O and C—H···O hydrogen bonds with H···A distance ranging from 1.55 (2) to 2.49 (2) Å (Table 1). Furthermore, in the crystalline network there is an intramolecular O—H···O hydrogen bond between phenolic OH and the carboxyl group (Fig. 2). In the crystal structure, C—H···π interactions (Table 1) [*Cg*1 is the centroid of C2–C7 benzene ring of H_2_sal] may further stabilize the structure. Above-mentioned van der Waals interactions lead to the formation and then expansion of a proton-transfer ligand.

### Experimental

By refluxing 0.14 mmol (0.025 g) H_2_sal and 0.14 mmol (0.025 g) Acr in 15 ml water for 3 h at 353 K, an orange solution was obtained. This solution gave orange needle-like crystal of the title compound after slow evaporation of the solvent at R.T.

### Refinement

H1 and H3–H7 atoms were positioned from Fourier map and other H atoms were positioned geometrically and allowed to ride during refinement isotropically. C—H distances are 0.93 Å for C(*sp*^2^) and and *U*_iso_ = p *U*_eq_(parent atom) [p = 1.2 for C(*sp*^2^)].

### Crystal data

**Table:**

C_13_H_10_N^+^·C_7_H_5_O_3_^−^	*F*(000) = 664
*M**_r_* = 317.33	*D*_x_ = 1.379 Mg m^−^^3^
Monoclinic, *P*2_1_/*c*	Mo *K*α radiation, λ = 0.71073 Å
Hall symbol: -P2ybc	Cell parameters from 1285 reflections
*a* = 7.128 (3) Å	θ = 2–25°
*b* = 9.472 (3) Å	µ = 0.09 mm^−^^1^
*c* = 22.637 (9) Å	*T* = 100 K
β = 91.449 (10)°	Prism, light-orange
*V* = 1527.9 (10) Å^3^	0.30 × 0.25 × 0.10 mm
*Z* = 4	

### Data collection

**Table:**

Bruker SMART APEXII CCD area-detector diffractometer	4488 independent reflections
Radiation source: fine-focus sealed tube	3161 reflections with *I* > 2σ(*I*)
graphite	*R*_int_ = 0.035
φ and ω scans	θ_max_ = 30.2°, θ_min_ = 1.8°
Absorption correction: multi-scan (*SADABS*; Bruker, 2001)	*h* = −10→8
*T*_min_ = 0.973, *T*_max_ = 0.991	*k* = −12→13
10437 measured reflections	*l* = −32→25

### Refinement

**Table:**

Refinement on *F*^2^	Primary atom site location: structure-invariant direct methods
Least-squares matrix: full	Secondary atom site location: difference Fourier map
*R*[*F*^2^ > 2σ(*F*^2^)] = 0.048	Hydrogen site location: inferred from neighbouring sites
*wR*(*F*^2^) = 0.128	H atoms treated by a mixture of independent and constrained refinement
*S* = 1.04	*w* = 1/[σ^2^(*F*_o_^2^) + (0.0581*P*)^2^ + 0.2765*P*] where *P* = (*F*_o_^2^ + 2*F*_c_^2^)/3
4488 reflections	(Δ/σ)_max_ < 0.001
241 parameters	Δρ_max_ = 0.37 e Å^−^^3^
0 restraints	Δρ_min_ = −0.24 e Å^−^^3^

### Special details

**Table:**

Geometry. All e.s.d.'s (except the e.s.d. in the dihedral angle between two l.s. planes) are estimated using the full covariance matrix. The cell e.s.d.'s are taken into account individually in the estimation of e.s.d.'s in distances, angles and torsion angles; correlations between e.s.d.'s in cell parameters are only used when they are defined by crystal symmetry. An approximate (isotropic) treatment of cell e.s.d.'s is used for estimating e.s.d.'s involving l.s. planes.
Refinement. Refinement of *F*^2^ against ALL reflections. The weighted *R*-factor *wR* and goodness of fit *S* are based on *F*^2^, conventional *R*-factors *R* are based on *F*, with *F* set to zero for negative *F*^2^. The threshold expression of *F*^2^ > σ(*F*^2^) is used only for calculating *R*-factors(gt) *etc*. and is not relevant to the choice of reflections for refinement. *R*-factors based on *F*^2^ are statistically about twice as large as those based on *F*, and *R*- factors based on ALL data will be even larger.

### Fractional atomic coordinates and isotropic or equivalent isotropic displacement parameters (Å^2^)

**Table:**

	*x*	*y*	*z*	*U*_iso_*/*U*_eq_	
C1	0.74651 (19)	0.32545 (14)	0.35776 (6)	0.0186 (3)	
C2	0.73287 (18)	0.43221 (14)	0.30953 (6)	0.0163 (3)	
C3	0.89699 (18)	0.48889 (14)	0.28532 (6)	0.0183 (3)	
C4	0.8833 (2)	0.59342 (15)	0.24213 (7)	0.0218 (3)	
C5	0.7091 (2)	0.63934 (16)	0.22190 (7)	0.0228 (3)	
C6	0.5458 (2)	0.58278 (16)	0.24467 (6)	0.0212 (3)	
C7	0.5587 (2)	0.47998 (15)	0.28815 (6)	0.0186 (3)	
C8	0.70546 (18)	0.32647 (15)	0.01980 (6)	0.0169 (3)	
C9	0.76980 (18)	0.40801 (15)	0.06919 (6)	0.0171 (3)	
C10	0.81472 (18)	0.54997 (15)	0.06022 (6)	0.0181 (3)	
H10	0.8588	0.6041	0.0919	0.022*	
C11	0.79458 (18)	0.61143 (15)	0.00465 (6)	0.0173 (3)	
C12	0.72746 (17)	0.52574 (15)	−0.04321 (6)	0.0171 (3)	
C13	0.70495 (19)	0.58406 (16)	−0.10047 (6)	0.0203 (3)	
H13	0.6604	0.5286	−0.1317	0.024*	
C14	0.74913 (19)	0.72240 (16)	−0.10951 (7)	0.0236 (3)	
H14	0.7341	0.7608	−0.1471	0.028*	
C15	0.8176 (2)	0.80885 (16)	−0.06248 (7)	0.0237 (3)	
H15	0.8473	0.9028	−0.0697	0.028*	
C16	0.84006 (19)	0.75551 (15)	−0.00700 (7)	0.0207 (3)	
H16	0.8852	0.8130	0.0235	0.025*	
C17	0.78718 (19)	0.34000 (16)	0.12514 (6)	0.0210 (3)	
H17	0.8286	0.3909	0.1581	0.025*	
C18	0.74345 (19)	0.20068 (16)	0.13068 (7)	0.0228 (3)	
H18	0.7544	0.1576	0.1675	0.027*	
C19	0.6817 (2)	0.12079 (16)	0.08115 (7)	0.0236 (3)	
H19	0.6535	0.0256	0.0858	0.028*	
C20	0.66274 (19)	0.18115 (15)	0.02653 (6)	0.0203 (3)	
H20	0.6224	0.1277	−0.0058	0.024*	
N1	0.68547 (15)	0.38796 (13)	−0.03385 (5)	0.0176 (2)	
O1	0.90825 (14)	0.28524 (12)	0.37528 (5)	0.0275 (3)	
O2	0.59605 (14)	0.28037 (11)	0.37986 (4)	0.0220 (2)	
O3	1.06952 (14)	0.44374 (11)	0.30336 (5)	0.0261 (3)	
H1	0.647 (3)	0.324 (2)	−0.0700 (10)	0.053 (6)*	
H3	1.044 (3)	0.376 (3)	0.3363 (11)	0.072 (8)*	
H4	0.994 (3)	0.634 (2)	0.2274 (8)	0.034 (5)*	
H5	0.699 (2)	0.7128 (19)	0.1916 (8)	0.029 (5)*	
H6	0.421 (2)	0.6185 (18)	0.2298 (8)	0.028 (5)*	
H7	0.447 (2)	0.4414 (16)	0.3052 (7)	0.018 (4)*	

### Atomic displacement parameters (Å^2^)

**Table:**

	*U*^11^	*U*^22^	*U*^33^	*U*^12^	*U*^13^	*U*^23^
C1	0.0232 (7)	0.0150 (6)	0.0173 (6)	0.0020 (5)	−0.0033 (5)	−0.0012 (5)
C2	0.0197 (7)	0.0127 (6)	0.0163 (6)	0.0005 (5)	−0.0011 (5)	−0.0011 (5)
C3	0.0179 (6)	0.0156 (6)	0.0214 (7)	0.0018 (5)	−0.0011 (5)	−0.0039 (5)
C4	0.0241 (7)	0.0174 (7)	0.0241 (8)	−0.0019 (5)	0.0046 (6)	−0.0002 (6)
C5	0.0320 (8)	0.0176 (7)	0.0189 (7)	0.0014 (6)	0.0013 (6)	0.0006 (6)
C6	0.0240 (7)	0.0203 (7)	0.0191 (7)	0.0041 (6)	−0.0035 (5)	0.0001 (5)
C7	0.0194 (7)	0.0177 (7)	0.0185 (7)	0.0007 (5)	−0.0010 (5)	−0.0008 (5)
C8	0.0128 (6)	0.0197 (7)	0.0182 (7)	0.0010 (5)	0.0010 (5)	−0.0011 (5)
C9	0.0135 (6)	0.0209 (7)	0.0168 (7)	0.0014 (5)	−0.0004 (5)	−0.0025 (5)
C10	0.0150 (6)	0.0202 (7)	0.0189 (7)	0.0011 (5)	−0.0016 (5)	−0.0043 (5)
C11	0.0132 (6)	0.0185 (7)	0.0202 (7)	0.0017 (5)	0.0000 (5)	−0.0019 (5)
C12	0.0125 (6)	0.0196 (7)	0.0193 (7)	0.0021 (5)	0.0007 (5)	−0.0009 (5)
C13	0.0179 (7)	0.0253 (7)	0.0177 (7)	0.0016 (5)	−0.0006 (5)	−0.0011 (6)
C14	0.0196 (7)	0.0282 (8)	0.0229 (7)	0.0042 (6)	0.0001 (5)	0.0055 (6)
C15	0.0206 (7)	0.0188 (7)	0.0315 (8)	0.0017 (5)	−0.0001 (6)	0.0028 (6)
C16	0.0177 (7)	0.0183 (7)	0.0262 (8)	0.0008 (5)	−0.0010 (5)	−0.0027 (6)
C17	0.0191 (7)	0.0257 (8)	0.0181 (7)	0.0014 (5)	−0.0007 (5)	−0.0019 (6)
C18	0.0211 (7)	0.0268 (8)	0.0206 (7)	0.0012 (6)	0.0006 (5)	0.0039 (6)
C19	0.0217 (7)	0.0210 (7)	0.0282 (8)	−0.0005 (5)	0.0016 (6)	0.0013 (6)
C20	0.0183 (7)	0.0203 (7)	0.0225 (7)	−0.0019 (5)	0.0003 (5)	−0.0033 (5)
N1	0.0158 (5)	0.0195 (6)	0.0176 (6)	0.0008 (4)	0.0000 (4)	−0.0032 (5)
O1	0.0222 (5)	0.0288 (6)	0.0311 (6)	0.0049 (4)	−0.0045 (4)	0.0095 (5)
O2	0.0231 (5)	0.0232 (5)	0.0197 (5)	−0.0012 (4)	−0.0018 (4)	0.0050 (4)
O3	0.0178 (5)	0.0223 (6)	0.0381 (7)	0.0019 (4)	−0.0009 (4)	0.0022 (5)

### Geometric parameters (Å, °)

**Table:**

C1—O1	1.2681 (17)	C11—C12	1.4268 (19)
C1—O2	1.2688 (17)	C11—C16	1.429 (2)
C1—C2	1.4895 (19)	C12—N1	1.3568 (19)
C2—C7	1.3966 (19)	C12—C13	1.414 (2)
C2—C3	1.4105 (19)	C13—C14	1.364 (2)
C3—O3	1.3551 (17)	C13—H13	0.9300
C3—C4	1.393 (2)	C14—C15	1.420 (2)
C4—C5	1.382 (2)	C14—H14	0.9300
C4—H4	0.943 (18)	C15—C16	1.359 (2)
C5—C6	1.392 (2)	C15—H15	0.9300
C5—H5	0.979 (18)	C16—H16	0.9300
C6—C7	1.386 (2)	C17—C18	1.362 (2)
C6—H6	1.002 (17)	C17—H17	0.9300
C7—H7	0.966 (16)	C18—C19	1.414 (2)
C8—N1	1.3511 (18)	C18—H18	0.9300
C8—C20	1.419 (2)	C19—C20	1.366 (2)
C8—C9	1.4252 (19)	C19—H19	0.9300
C9—C10	1.398 (2)	C20—H20	0.9300
C9—C17	1.424 (2)	N1—H1	1.05 (2)
C10—C11	1.3901 (19)	O3—H3	1.01 (3)
C10—H10	0.9300		
			
O1—C1—O2	123.12 (13)	C12—C11—C16	118.46 (13)
O1—C1—C2	118.37 (12)	N1—C12—C13	119.90 (13)
O2—C1—C2	118.51 (12)	N1—C12—C11	119.98 (12)
C7—C2—C3	118.74 (13)	C13—C12—C11	120.12 (13)
C7—C2—C1	121.00 (12)	C14—C13—C12	119.43 (13)
C3—C2—C1	120.25 (12)	C14—C13—H13	120.3
O3—C3—C4	118.87 (13)	C12—C13—H13	120.3
O3—C3—C2	121.19 (13)	C13—C14—C15	121.19 (14)
C4—C3—C2	119.94 (13)	C13—C14—H14	119.4
C5—C4—C3	120.18 (13)	C15—C14—H14	119.4
C5—C4—H4	120.2 (11)	C16—C15—C14	120.57 (14)
C3—C4—H4	119.6 (11)	C16—C15—H15	119.7
C4—C5—C6	120.56 (14)	C14—C15—H15	119.7
C4—C5—H5	120.5 (10)	C15—C16—C11	120.24 (14)
C6—C5—H5	119.0 (10)	C15—C16—H16	119.9
C7—C6—C5	119.50 (14)	C11—C16—H16	119.9
C7—C6—H6	121.3 (10)	C18—C17—C9	120.34 (13)
C5—C6—H6	119.2 (10)	C18—C17—H17	119.8
C6—C7—C2	121.07 (13)	C9—C17—H17	119.8
C6—C7—H7	120.7 (9)	C17—C18—C19	120.86 (14)
C2—C7—H7	118.2 (9)	C17—C18—H18	119.6
N1—C8—C20	119.78 (12)	C19—C18—H18	119.6
N1—C8—C9	119.74 (13)	C20—C19—C18	121.03 (14)
C20—C8—C9	120.47 (13)	C20—C19—H19	119.5
C10—C9—C17	123.32 (13)	C18—C19—H19	119.5
C10—C9—C8	118.52 (13)	C19—C20—C8	119.13 (13)
C17—C9—C8	118.15 (13)	C19—C20—H20	120.4
C11—C10—C9	121.02 (13)	C8—C20—H20	120.4
C11—C10—H10	119.5	C8—N1—C12	122.44 (12)
C9—C10—H10	119.5	C8—N1—H1	118.3 (12)
C10—C11—C12	118.28 (13)	C12—N1—H1	119.1 (12)
C10—C11—C16	123.25 (13)	C3—O3—H3	104.2 (14)
			
O1—C1—C2—C7	179.63 (13)	C10—C11—C12—N1	−0.11 (18)
O2—C1—C2—C7	−1.2 (2)	C16—C11—C12—N1	179.08 (12)
O1—C1—C2—C3	−1.5 (2)	C10—C11—C12—C13	−179.99 (12)
O2—C1—C2—C3	177.62 (12)	C16—C11—C12—C13	−0.79 (18)
C7—C2—C3—O3	−178.30 (12)	N1—C12—C13—C14	−179.43 (12)
C1—C2—C3—O3	2.8 (2)	C11—C12—C13—C14	0.44 (19)
C7—C2—C3—C4	1.8 (2)	C12—C13—C14—C15	0.1 (2)
C1—C2—C3—C4	−177.04 (13)	C13—C14—C15—C16	−0.3 (2)
O3—C3—C4—C5	178.54 (13)	C14—C15—C16—C11	−0.1 (2)
C2—C3—C4—C5	−1.6 (2)	C10—C11—C16—C15	179.77 (13)
C3—C4—C5—C6	0.5 (2)	C12—C11—C16—C15	0.62 (19)
C4—C5—C6—C7	0.2 (2)	C10—C9—C17—C18	−179.08 (13)
C5—C6—C7—C2	0.0 (2)	C8—C9—C17—C18	0.14 (19)
C3—C2—C7—C6	−1.1 (2)	C9—C17—C18—C19	0.5 (2)
C1—C2—C7—C6	177.80 (13)	C17—C18—C19—C20	−0.5 (2)
N1—C8—C9—C10	−1.41 (18)	C18—C19—C20—C8	−0.2 (2)
C20—C8—C9—C10	178.42 (12)	N1—C8—C20—C19	−179.31 (12)
N1—C8—C9—C17	179.33 (12)	C9—C8—C20—C19	0.86 (19)
C20—C8—C9—C17	−0.84 (18)	C20—C8—N1—C12	−178.74 (12)
C17—C9—C10—C11	−179.78 (12)	C9—C8—N1—C12	1.09 (19)
C8—C9—C10—C11	1.00 (19)	C13—C12—N1—C8	179.55 (12)
C9—C10—C11—C12	−0.26 (19)	C11—C12—N1—C8	−0.32 (19)
C9—C10—C11—C16	−179.41 (13)		

### Hydrogen-bond geometry (Å, °)

**Table:**

*D*—H···*A*	*D*—H	H···*A*	*D*···*A*	*D*—H···*A*
N1—H1···O1^i^	1.05 (2)	2.49 (2)	3.100 (2)	116.4 (15)
N1—H1···O2^i^	1.05 (2)	1.55 (2)	2.5887 (19)	174.8 (18)
O3—H3···O1	1.00 (3)	1.58 (2)	2.5141 (19)	153 (2)
C10—H10···O1^ii^	0.93	2.49	3.294 (2)	145
C18—H18···O3^iii^	0.93	2.46	3.135 (2)	129
C14—H14···Cg1^iv^	0.93	2.76	3.644 (2)	159
C17—H17···Cg1	0.93	2.91	3.716 (2)	146

Symmetry codes: (i) *x*, −*y*+1/2, *z*−1/2; (ii) −*x*+2, *y*+1/2, −*z*+1/2; (iii) −*x*+2, *y*−1/2, −*z*+1/2; (iv) *x*, −*y*+3/2, *z*−1/2.
